# Angioedema: A Possible Complication of Amlodipine Use

**DOI:** 10.7759/cureus.42202

**Published:** 2023-07-20

**Authors:** Thomas Russo, Sarkis Kouyoumjian, Hithem Fargaly

**Affiliations:** 1 Emergency Medicine, Wayne State University School of Medicine, Detroit, USA; 2 Emergency Medicine, Wayne State University Detroit Medical Center, Detroit, USA; 3 Internal Medicine, Wayne State University Detroit Medical Center, Detroit, USA

**Keywords:** verapamil, diltiazem, nifedipine, dihydropyridine calcium channel blocker, facial angioedema, calcium channel blocker, amlodipine, drug-induced angioedema

## Abstract

Angioedema is a documented but uncommon adverse effect of dihydropyridine calcium channel blockers such as amlodipine. We present the case of a 38-year-old man who presented to the emergency department (ED) with severe swelling of his upper lip that had begun earlier in the day. His medical history was notable for hypertension treated with amlodipine; his only other medication was a multivitamin. The patient denied any known drug allergies, new foods, insect bites, recent travel, or sick contacts. Physical examination showed hypertension and massive edema isolated to the upper lip; it was otherwise unremarkable. Laboratory results showed no abnormalities aside from a slight normocytic anemia. The patient was diagnosed with angioedema, with amlodipine suspected as the cause. Amlodipine was discontinued and treatment was initiated with IV glucocorticoids and diphenhydramine. The swelling improved steadily over the next 36 hours and the patient was discharged on hospital day 3.

## Introduction

Angioedema is characterized by an accumulation of fluid in the subcutaneous or submucosal interstitium and can be life-threatening when it involves the upper airway [1]. Its pathogenesis can involve a broad variety of etiologies, and drug reaction to an angiotensin-converting enzyme (ACE) inhibitor is a well-known cause [2].

Calcium channel blockers are a rare cause of angioedema [3]. Cases involving nifedipine, verapamil, and diltiazem have been documented starting in the mid-1980s [4-8]. More recently, a handful of case reports have described instances of angioedema caused by amlodipine [3,9-14]. The mainstay of treatment, as for all causes of angioedema, is discontinuation of the causative agent and careful management of the airway with intubation if necessary [1]. Beyond these basics, however, the management of angioedema caused by amlodipine is challenging due to its rarity and limited coverage in the medical literature.

We present a case report of a 38-year-old man who presented with massive swelling of his upper lip due to angioedema. Based on a careful review of his medications and detailed history that excluded other possible causes, amlodipine was identified as the likely causative agent. His treatment course was successful and uncomplicated, and as such it provides a reasonable guide for the management of this condition.

## Case presentation

A 38-year-old male presented to the emergency department (ED) in the early evening complaining of upper lip swelling which began earlier in the day and progressively worsened. The patient denied any difficulty breathing, trouble swallowing, tongue swelling, or rash. His only medications were amlodipine, started six to eight weeks ago, and a multivitamin. Other than a remote history of a possible allergic reaction to a bee sting, he denied any known allergies and specifically denied recent bee stings or other insect bites. He further denied recent sick contacts, new foods, or other exposures. He endorsed alcohol consumption one to two times per month and one to one-and-a-half packs per day of cigarettes. He denied any recreational drug use. At the time of admission, the patient was working as a truck driver making deliveries to numerous warehouses each day. He denied any known exposures to industrial chemicals or other agents in the course of his job. The history provided by the patient was found to be consistent with his electronic medical record.

On physical examination, the patient was hypertensive at 173/95 but hemodynamically stable, afebrile, and in no acute distress. Head, eyes, ears, nose, and throat examination showed swelling isolated to the upper lip. There was no oropharyngeal, uvular, submandibular, or sublingual swelling and no dysphonia. The posterior oropharynx was unremarkable. The patient was tolerating secretions and showed no signs of airway compromise. Lungs were clear to auscultation bilaterally, the heart had a normal rate and rhythm, and the skin exam showed no rash. Examination of other organ systems revealed no abnormal findings. Routine laboratory values were unremarkable. Due to low clinical suspicion, no imaging studies were obtained.

**Figure 1:**
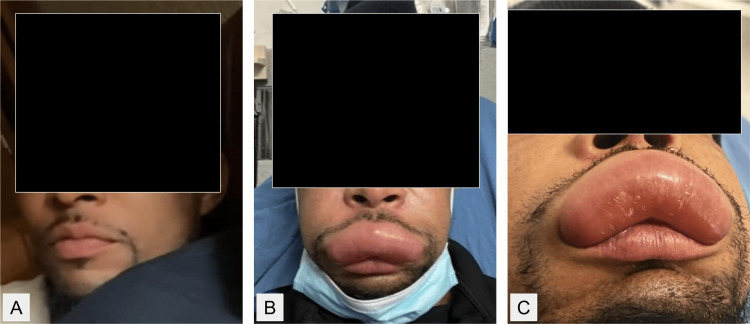
Images comparing a recent photo of the patient (A) with his appearance when he presented to the emergency department at roughly 7:00 pm (B and C).

**Figure 2:**
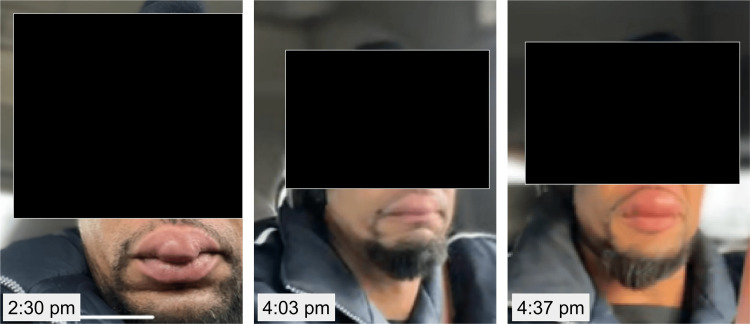
Progression of the angioedema. Images annotated with the corresponding time.

Photographs were taken in the ED to document the patient’s presentation (Figure 1). In addition, the patient made several video calls to his partner throughout the day, and she took several screenshots which she shared with the patient’s care team (Figure 2). These images show the progression of the edema as it began on the medial aspect of the upper lip and then progressed laterally over several hours.

Based on the patient’s presentation, the differential diagnosis included both idiopathic angioedema and a reaction to a drug, insect bite, food product, or environmental toxin. The majority of these were excluded by a careful history. A tentative diagnosis of angioedema induced by amlodipine was made based on the fact that this drug was recently initiated. Amlodipine was discontinued and the patient was given IV dexamethasone 10 mg, IV diphenhydramine 50 mg, IV famotidine 20 mg, and IV tranexamic acid 1000 mg, with no improvement over the next 6 hours. He was then given an additional dose of IV famotidine 20 mg and started on methylprednisolone 60 mg once every 6 hours.

When the patient was seen the next morning in the ED, roughly 12 hours after presentation, the swelling of his upper lip had decreased significantly. He was admitted to the medicine service for observation. Diphenhydramine and methylprednisolone were both continued at the same dosages, once every 6 hours. Other medications started in the ED were discontinued. The patient’s condition continued to improve, with full resolution of the angioedema by mid-afternoon on hospital day 2. Diphenhydramine and methylprednisolone were discontinued at that time. The patient remained stable for the rest of his hospital course with no recurrence of the angioedema. He was discharged early on hospital day 3 with prescriptions for oral cetirizine 10 mg daily, oral prednisone 50 mg daily for three days, and an Epi-pen. In addition, amlodipine was replaced with oral chlorthalidone 25 mg daily. He was instructed to follow up with his PCP within one week. Amlodipine was added as a new allergy in the patient’s electronic medical record, although it should be noted that the mechanism of this adverse effect is unknown.

During the patient’s hospital stay, an extensive review of his history was undertaken to rule out other possible causes of angioedema. He confirmed that he was taking no medications other than amlodipine and a multivitamin. Ultimately no alternative cause was found.

Retrospectively, a Naranjo score was calculated to evaluate the likelihood this reaction was caused by amlodipine. This score is computed based on the answers to 10 questions and stratifies the likelihood a specific drug caused an observed reaction into four categories: definite (score ≥9), probable (scores of 5-8), possible (scores of 1-4), and unlikely (score ≤0) [15]. A Naranjo score of 7 was calculated for this adverse reaction, indicating that amlodipine is a probable cause. It should be noted, however, that idiopathic angioedema cannot be ruled out.

## Discussion

The rarity of amlodipine-induced angioedema makes its management challenging. A review of the literature yielded five detailed case reports involving amlodipine either alone or in combination with other calcium channel blockers [9-13]. In addition, a number of likely or possible cases were found that lacked significant detail. Three come from a large clinical trial of antihypertensive medications (the ALLHAT trial) [14] and 14 from a retrospective case series at a single institution [3]. The cases from the ALLHAT trial represent 0.03% of patients randomized to the amlodipine group and this figure provides the best existing estimate of the incidence of amlodipine-induced angioedema in the general population.

In the five detailed case reports, the only treatment element consistently used in every case was discontinuation of amlodipine. This was the only treatment employed in two cases [11,13]. In the other cases, management included diphenhydramine, hydrocortisone, and ranitidine [12]; dexamethasone, epinephrine, and supplemental oxygen [10]; and “steroids and antihistamines” [9]. The angioedema resolved in all five cases, generally in 2-3 days. This suggests that in cases of suspected amlodipine-induced angioedema, discontinuation of the drug is the most important step. The benefit of pharmacotherapy is unclear as the mechanism of calcium-channel-blocker-induced angioedema is unknown [9]. The use of steroids, antihistamines, and epinephrine is presumably due to their utility in treating hypersensitivity reactions. In our case, accelerated resolution of the angioedema coincided with the switch from IV dexamethasone to IV methylprednisolone. It is unclear whether the relationship is causal, and it should be noted that antihistamines (diphenhydramine and famotidine) were given concurrently with both steroids. Since the benefits of steroids, antihistamines, and epinephrine are not established, their use must be based on clinical judgment. It should be noted that this treatment approach differs from the one used for ACE inhibitor-induced angioedema, which targets bradykinin using fresh frozen plasma and/or a bradykinin blocker such as icatibant [16].

A final aspect of this case that is notable is the significant likelihood that amlodipine is in fact responsible for this adverse reaction. While the case reports discussed above strongly suggest that amlodipine is a possible cause of angioedema, this link is difficult to establish definitively because 1) patients on calcium channel blockers such as amlodipine are often prescribed multiple other medications, and 2) no allergy testing protocol exists for amlodipine [9]. The lack of other prescription drugs or known alternative causes of angioedema, in this case, adds weight to the evidence that angioedema can be induced by amlodipine, although it should be emphasized that idiopathic angioedema cannot be ruled out.

## Conclusions

Prompt management of angioedema is critical, particularly in cases involving the upper airway. Among the many possible causes of this condition, amlodipine is documented but uncommon. As a result, the identification of this drug as the culprit can be delayed or missed. In addition to the more common causes, amlodipine, along with other calcium channel blockers, should be considered as a possible etiology in cases of angioedema.
